# Development and reliability of a self-report questionnaire to examine children's perceptions of the physical activity environment at home and in the neighbourhood

**DOI:** 10.1186/1479-5868-3-16

**Published:** 2006-07-17

**Authors:** Clare Hume, Kylie Ball, Jo Salmon

**Affiliations:** 1 Centre for Physical Activity and Nutrition Research, Deakin University, 221 Burwood Hwy, Burwood, Victoria 3125, Australia

## Abstract

**Background:**

Environmental factors are increasingly being implicated as key influences on children's physical activity. Few studies have comprehensively examined children's perceptions of their environment, and there is a paucity of literature on acceptable and reliable scales for measuring these. This study aimed to develop and test the acceptability and reliability of a scale which examined a broad range of environmental perceptions among children.

**Methods:**

Based on constructs from ecological models, a survey incorporating items on children's perceptions of the physical and social environment at home and in the neighbourhood was developed. This was administered on two occasions, nine days apart, to a sample of 39 children aged 11 years (54% boys), attending a metropolitan Australian elementary school. The acceptability of the survey was determined by the proportion of missing responses to each item. The test-retest reliability of individual items, scores and scales were determined using Kappa statistics and percent agreement for categorical variables, and intraclass correlation coefficients (ICC) for continuous variables.

**Results:**

There were few missing responses to each question, with only 4% of all responses missing. Although some Kappa values were low, all categorical variables showed acceptable reliability when examined for percent agreement between test and retest (range 68%–100% agreement). Continuous variables all showed moderate to good ICC values (range 0.72–0.92).

**Conclusion:**

Findings suggest this questionnaire is reliable and acceptable to children for assessing environmental perceptions relevant to physical activity among 11-year-old children.

## Background

The physical and psychological health benefits of being physically active are well known [1]; however secular declines in children's physical activity are evident [2]. In order to develop effective strategies to arrest these declines, understanding factors that influence children's activity is important. Ecological models posit that there are multiple levels of influence on physical activity, including intrapersonal, social and physical environmental factors [3]. Intrapersonal factors have been widely investigated, and although measures are well conceptualised and established, these factors appear only to explain part of the variance in children's activity [4].

The home and the local neighbourhood are two important settings for children's physical activity. Studies among adults have commonly defined the physical environment in terms of accessibility and availability of destinations and facilities, aesthetics, and safety [5]. Few studies have attempted to define, operationalise or measure these among children, and none have examined the psychometric properties of such measures. Children's social environment has typically been defined in terms of the proximal social influences (e.g. parental support), and measures are well established [6]. However, with few exceptions [7], almost no studies have assessed children's broader social environment (e.g. the local neighbourhood).

The aim of this study was to develop and test the reliability and acceptability of an instrument to assess a broad range of environmental perceptions that might predict physical activity among children, particularly the physical and social environments at home and in the neighbourhood.

## Participants and methods

A convenience sample of Grade 5 and 6 children attending a metropolitan Melbourne elementary school participated (21 boys, 18 girls, aged 11.1 ± 0.7 years).

### Measures

Children were asked to report their perceptions of the physical and social environments at home and in the neighbourhood. All items are shown in Table 1.

**Table 1:** The psychometric properties and the number of missing responses to items examining children's perceptions of the physical activity environment

**The home environment**	**ICC**		**CI**	# of missing responses/39*
**The physical environment at home**	.89		.80–.94	1

Please tell us about your yard.				
We have:				
No yard at all				
A small yard (eg. a unit)				
A medium yard (eg. A normal block of land)				
A large yard (eg. 1/4 acre or more)				

	**Kappa (CI)**	**p**	**% agreement**	# of missing responses/39*

Which of the following do you have in your home, or outside in your yard? (Have at home Y/N)				
Outside:				
Front fence	.74 (-.13, .29)	.0001	87	0
Swimming pool	.91 (-.10, .26)	.0001	85	0
Trampoline	1.0 (n/a)	.0001	100	0
Basketball ring	.87 (-.10, .25)	.0001	95	0
Covered area outdoors	.13 (-.35, .40)	.37	82	0
Paved area outdoors	.71 (-.17, .36)	.0001	90	0
Sandpits, swings or play equipment	.84 (-.12, .30)	.0001	95	0
				
Think about the last month. How often did you do the following activities at home? (Have at home Y/N)				
Played with bats/racquets/golf clubs	.35 (-.31, .45)	.03	82	2
Played with balls	1.0 (n/a)	.0001	100	2
Rode my bike	.37 (-.43, .63)	.003	92	1
Went rollerblades	.58 (-.20, .36)	.0001	81	2
Rode my skateboard	.68 (-.15, .31)	.0001	84	1
Jumped with my skipping rope	.45 (-.22, .35)	.004	74	0
Rode my scooter	.74 (-.13, .29)	.0001	87	0
Played with toys that I run around with (e.g. frisbees, water pistols)	.06 (-.22, .23)	.59	68	2
Played outside with my pet	1.0 (n/a)	.0001	100	4
				
**The social environment at home**				
				
Think about the last month. How often were the following people physically active with you? (Have at home Y/N)				
Whole family was active together	.30 (-.27, .36)	.06	69	4
Was active with Father	.23 (-.33, .42)	.20	73	6
Was active with Mother	.58 (-.23, .43)	.0001	86	3
Was active with grandparents	.65 (.21, .42)	.001	85	13
Was active with siblings	.26 (-.45, .59)	.15	87	8
Was active with friends	.53 (-.29, .51)	.002	89	4
				
Think about the last month. Did a friend or family member offer you encouragement or support to be physically active? (Have at home Y/N)				
Received offers to be physically active with a family member	.16 (-.34, .39)	.32	74	4
Received encouragement for physical activity from a family member	1.0 (n/a)	.0001	100	5
Received offers to be physically active with friends	.65 (-.19, .37)	.0001	87	1
Received encouragement for physical activity from friends	.42 (-.23, .36)	.01	71	4

**The neighbourhood environment**	**Kappa (CI)**	**p**	**% agreement**	# of missing responses/39*

**The physical environment in the neighbourhood**				
Think about the last month. How often did you walk or ride your bike to the following places? (Have at home Y/N)				
My friends' houses	.69 (-.19, .38)	.0001	89	1
The post box	.13 (-.35, .41)	.39	83	1
Public transport	-.08 (-.08, .08)	.61	83	3
School	.54 (-.29, .51)	.001	89	1
The shops/milk bar	1.0 (n/a)	.0001	100	2
The lake	.60 (-.18, .33)	.0001	81	2
The golf course	.60 (-.18, .33)	.0001	81	4
Bike/walking tracks or trails	.42 (-.25, .39)	.007	78	2
The local basketball courts	.41 (-.31, .47)	.01	84	1
The local oval	.54 (-.29, .21)	.001	89	3
The local park	.72 (-.23, .49)	.0001	94	3
The local recreation centre	.54 (-.21, .38)	.001	82	1
The local shopping centre	.57 (-.20, .36)	.0001	82	1
The local swimming pool	.51 (-.20, .34)	.001	76	2
The local tennis courts	.43 (-.22, .35)	.003	76	1
				
Which of the following statements are true about the area you live in? (Have at home Y/N)				
**Aesthetics – pleasing aesthetics**				
There are lots of nice houses	.77 (-.18, .42)	.0001	95	2
It's a nice and quiet place to live	.54 (-.28, .50)	.001	89	2
The houses have nice gardens	.53 (-.41, .59)	.001	92	1
**Aesthetics – incivilities**				
There is lots of litter and rubbish	-.03 (-.04, .04)	.87	92	2
There is lots of graffiti	1.0 (n/a)	.0001	100	1
**Safety – general **				
It's easy to walk/cycle around	1.0 (n/a)	.0001	100	3
It's a safe area to walk/cycle	.53 (-.34, .60)	.001	92	2
It's safe to walk/cycle to school	.54 (-.22, .38)	.001	82	1
**Safety – traffic/road **				
The roads are safe	.53 (-.26, .46)	.001	86	3
Feel safe crossing the road	84 (-.17, .43)	.0001	97	1
There is heavy traffic	.53 (-.34, .59)	.001	92	1
**Safety – personal**				
Worried about dogs roaming the streets	1.0 (n/a)	.0001	100	2
Worried about strangers	.53 (-.34, .59)	.001	92	1
Worried about older kids hanging around	-.07 (-.07, .06)	.67	87	1
				
**The social environment in the neighbourhood**				
Which of the following statements are true about the area you live in? (Have at home Y/N)				
I have many friends in my area	.79 (-.14, .32)	.0001	92	1
I have friends who live within a walking or cycling distance from my house	.87 (-.10, .26)	.0001	95	1
I have children living next door or in street who I can play with	.63 (-.18, .35)	.0001	84	2
I know many people in my area	.62 (-.21, .40)	.0001	87	1
There are lots of children around to play with	.38 (-.23, .33)	.01	68	1
I know all of my neighbours quite well	.58 (-.18, .33)	.0001	79	2
I know some of my neighbours quite well	.42 (-.28, .44)	.001	81	3

* The total number of missing responses between test and retest from 39 participants

Items examining the size of the yard (1 item) and physical activity opportunities at home (16 items) were adapted from a published parent proxy-report survey examining children's leisure activities [8]. Responses were either dichotomous, or on a seven-point scale (later dichotomised), indicating whether the child did or did not have these items at home. A summed score indicating the number of opportunities for physical activity at home was created (range 0–16).

Ten items assessed the home social environment. Six items on family physical activity were adapted from a parent proxy-report survey [8]. Responses ranged from 'never' to 'daily', and were collapsed into no (never) or yes (all other options) response options. Four items on encouragement and support for activity were derived from previous study of psychosocial influences on children's physical activity [6]. Response options ranged from 'never' to 'daily', which were collapsed into yes/no categories. A scale evaluating the overall home social environment was generated by summing the positive (yes) responses to each of the 10 items (range 0–10), with a higher score indicating a more positive perceived home social environment.

Children's perceptions of the neighbourhood physical environment (access to destinations, aesthetic, and safety characteristics) were assessed with 29 items. Access (by active transport) to 15 neighbourhood destinations was assessed using items from a study examining Australian children's leisure activities [8]. Responses were on a seven-point scale with options ranging from never to daily as well as 'it's not within walking/cycling distance'. Based on this last option, responses were collapsed into two categories; 'can't access' and 'can access'. A score indicating the total number of neighbourhood destinations accessible by active transport was created by summing the positive responses to each item (range 0–15).

Fourteen items were used to assess children's perceptions of the neighbourhood aesthetic and safety characteristics, adapted from questions used in a study among adults [9]. Each response was dichotomised ('yes', or 'no/don't know'). Overall aesthetic and safety scales were developed by reverse coding negatively-worded items and summing the 'yes' responses. A higher score on these scales indicated more positive perceptions of neighbourhood aesthetics (range 0–5) and safety characteristics (range 0–9).

Questions assessing children's perceptions of the neighbourhood social environment were developed specifically for use in this study. Seven items (dichotomised to 'yes' or 'no/don't know' response options) assessed children's perceptions of the social connections in their neighbourhood. An overall neighbourhood social environment scale was generated by summing the positive responses, with a higher score indicating more positive perceptions.

### Survey administration

All questions were completed on two occasions (Times 1 and 2) up to nine days apart, during class time with researchers and the classroom teacher present.

### Statistical analyses

The survey was assessed for acceptability by calculating the proportion of missing responses to each item. Intra-class correlations (ICC's) were used to examine similarities between responses for continuous variables [10], while the Kappa statistic (κ) and percent agreement between responses (the proportion of participants grouped within the same response category for test and retest), were used to determine the repeatability of the categorical variables. Adequate test-retest reliability was defined as ICC ≥ 0.75 for continuous variables [11], and the strength of agreement between responses to categorical variables using κ was defined as poor to fair (0.00–0.40), moderate (0.41–0.60), substantial (0.61–0.80) and almost perfect (0.81–1.0) [12]. Significance (p-value) was set at 0.05, which indicated if the strength of agreement between responses was statistically significant according to κ. Percent agreement was also calculated since the stability of κ is dependent on the prevalence of responses to a question and when there is a low prevalence of one response, κ may be low [13]. Percent agreement values greater than 66% were classified as fair [14].

Internal reliability analyses (Cronbach's α) were performed on scaled responses to multiple items (e.g. the neighbourhood safety scale). Internal reliability was deemed acceptable if Cronbach's α was greater than 0.6 [11].

## Results

The survey appeared acceptable for children to complete, as they asked few questions, did not report any difficulties understanding the questions and only 11% of responses were missing (Table 1). The items with the most missing responses were those asking about physical activity with family members, particularly with grandparents (13/39 missing), suggesting these items may be difficult for children to complete (possibly reflecting the fact that some children may not have grandparents or siblings, or recall difficulties). On balance, the survey appears to be feasible and acceptable for children.

Results from test-retest reliability analyses are shown in Table 1. The home physical environment items (17 items) showed at least moderate reliability with the exception of two items (having a covered area outdoors and having active toys). All items showed at least fair agreement between test and retest. The physical activity opportunities at home score showed acceptable repeatability (ICC = 0.80, CI = 0.55–0.91). Of the 10 home social environment items, one (receiving offers for physical activity from family members) showed poor reliability according to κ (0.16); however all items assessing the home social environment showed at least fair agreement (69% or better) from test to re-test. The home social environment scale also showed adequate test-retest reliability (ICC = 0.84, CI = 0.56–0.94) and good internal reliability (Cronbach's α = 0.73).

The 29 neighbourhood physical environment items showed at least moderate reliability, with the exception of two access to destinations items (can access the post box and public transport), one aesthetic item (there is lots of litter and rubbish) and one safety item (worried about older kids hanging around), which showed poor κ values. All items showed at least fair agreement (greater than 75%) between test and retest. The total number of accessible destinations in the neighbourhood score showed excellent test-retest reliability (ICC = 0.84, CI = 0.66–0.93) and the aesthetics and safety scales showed acceptable test-retest reliability (ICC = 0.72, CI = 0.45–0.86; ICC = 0.88, CI = 0.76–0.94 respectively). Internal reliability analyses showed that while the safety scale was acceptable (Cronbach's α = 0.65), the aesthetics scale was not (Cronbach's α = 0.43).

Of the seven items examining the neighbourhood social environment, only one showed less than moderate test-retest reliability (having lots of other children around to play with). All items showed at least fair agreement (68% or better) from test to retest. The neighbourhood social environment scale showed adequate test-retest reliability (ICC = 0.92, CI = 0.84–0.96) and good internal reliability (Cronbach's α = 0.77).

Although several items showed poor to moderate test-retest reliability according to κ, all items in the questionnaire showed at least fair agreement (greater than 68%) between test and retest.

## Conclusion

This study is the first to assess the test-retest reliability of a broad range of questions examining children's perceptions of their physical and social environment at home and in the neighbourhood. The questions were mostly found to be acceptable and appropriate for this age group. Although several items did have poor κ values, all items showed at least adequate agreement between test and retest. Further research may be required to design a questionnaire using language that may be more appropriate for children, to better define and operationalise the neighbourhood aesthetic environment for children, and to validate such measures of the environment. The results of this study provide some support for the use of these items when examining environmental determinants of physical activity among children.

## Competing interests

The author(s) declare that they have no competing interests.

## Authors' contributions

CH, KB and JS conducted the study, CH drafted the manuscript, and KB and JS contributed to the analysis, interpretation and writing. All authors read and approved the final manuscript.
